# Phenotypic Fosfomycin Susceptibility and Detection of Plasmid-Mediated Resistance Genes in Clinical Uropathogenic *Escherichia coli* Isolates

**DOI:** 10.3390/microorganisms14061291

**Published:** 2026-06-08

**Authors:** Eman E. Hegazy, Esraa A. Mohamed, Shaimaa S. E. Marey, Aya Abdelrahman, Aysha Femy, Esraa Atef, Asmaa K. Hammad, Amira E. Oraiby, Ahmed Mostafa Elgohary, Ahmed Metawee Elsefy, Ahmed Boshnak, Manar M. Emara

**Affiliations:** 1 Department of Medical Microbiology and Immunology, Faculty of Medicine, Tanta University, Tanta 31527, Egypt; shimaa.marey@med.tanta.edu.eg (S.S.E.M.); manaremara@med.tanta.edu.eg (M.M.E.); 2 Department of medicine, Cairo University, Cairo 11956, Egypt; ayanasser589@gmail.com; 3 General Medicine Practice Program, Batterjee Medical College, Dammam 31433, Saudi Arabia; aysha.femy@bmc.edu.sa (A.F.); esraa.salem@bmc.edu.sa (E.A.); asmaa.hammad@bmc.edu.sa (A.K.H.); 4 Department of Clinical Pathology, Faculty of Medicine, Tanta University, Tanta 31527, Egypt; amira.oreiby@gmail.com (A.E.O.); ahmed.elgohary@med.edu.eg (A.M.E.); 5 Department of Internal Medicine, Faculty of Medicine, Tanta University, Tanta 31527, Egypt; ahmedmetawea86@gmail.com; 6 Urology Department, Faculty of Medicine, Ain Shams University, Cairo 11591, Egypt; ahmedboshnak0@gmail.com

**Keywords:** urinary tract infections, *Escherichia coli*, fosfomycin resistance, *fos* genes

## Abstract

Urinary tract infections (UTIs), predominantly caused by *Escherichia coli*, constitute a major global health issue due to the escalating antimicrobial resistance. This study was designed to assess the in vitro susceptibility of fosfomycin among clinical uropathogenic *Escherichia coli* (UPEC) isolates and to detect plasmid-mediated *fosA3* and *fosC2* genes in those exhibiting fosfomycin resistance. A total of 158 non-duplicated UPEC strains were collected from urine samples of patients with UTIs. Antimicrobial susceptibility of these isolates was evaluated. Phenotypic detection of extended-spectrum β-lactamases (ESBL) and carbapenemase producers in UPEC was assessed. Identification of plasmid mediated *fosA3* and *fosC2* genes in those exhibiting fosfomycin resistance was carried out by PCR. A total of 72% of the isolates demonstrated multidrug resistance (MDR). Extensively drug-resistant (XDR) isolates represented 15%, while PDR isolates were rare (0.6%, 1/158). ESBLs were detected in 40% of the isolates, and 31% exhibited carbapenemase production. Fosfomycin resistance was detected in 9.5% of UPEC isolates, with the *fosA3* gene identified in 33% of these resistant strains, whereas *fosC2* gene was not identified in any isolate. Fosfomycin demonstrated considerable in vitro activity against carbapenemase-producing, ESBL-producing, and MDR isolates with susceptibility rates of 78%, 84%, and 97%, respectively. Fosfomycin resistance among UPEC isolates is emerging but still at a relatively low level of resistance. Continuous surveillance and antimicrobial stewardship are essential to preserve fosfomycin efficacy.

## 1. Introduction

Urinary tract infections (UTIs) are widespread bacterial infections globally, occurring in healthcare and community settings. They are among the leading causes of antimicrobial prescriptions, leading to significant healthcare costs [1,2]. The majority of UTIs, approximately 80–90%, are attributed to uropathogenic *Escherichia coli* (UPEC). Other clinically relevant pathogens include *Klebsiella pneumoniae*, *Proteus mirabilis*, *Enterococcus faecalis*, and *Staphylococcus saprophyticus* [3].

From a clinical perspective, UTIs are commonly classified into uncomplicated (uUTIs) or complicated (cUTIs) forms [4]. Current therapeutic guidelines for uUTIs recommend several medications, such as trimethoprim–sulfamethoxazole, nitrofurantoin, β-lactam antibiotics, and fluoroquinolones. Nevertheless, many uropathogens have developed significant resistance to these standard antimicrobials, leading to the emergence and spread of multidrug-resistant organisms (MDROs) [5].

In recent years, the management of UTIs has become increasingly challenging owing to the rising incidence of multidrug-resistant (MDR) and extended-spectrum β-lactamase (ESBL)-producing UPEC strains. These resistant phenotypes substantially limit the therapeutic options available and necessitate consideration of alternative treatment strategies [6]. Moreover, the rapid development of resistance to existing antibacterial agents, combined with a decline in the introduction of new medications, highlights the need to re-evaluate the reuse of older antibiotics [7].

Fosfomycin is a broad-spectrum bactericidal antibiotic that exerts its activity by inhibiting bacterial cell wall synthesis through irreversible inactivation of the MurA enzyme, which catalyzes a key step in peptidoglycan biosynthesis [8,9]. Fosfomycin resistance in *E. coli* can arise from various mechanisms, including reduced intracellular drug accumulation due to chromosomal mutations affecting the UhpT and GlpT transport systems, alterations in the MurA target site, and enzymatic drug inactivation mediated by plasmid-encoded *fos* genes, which encode enzymes that catalyze the conjugation of glutathione to fosfomycin, thereby rending the drug ineffective [10].

In Enterobacterales, the characterized *fos* genes include *fosA1* to *fosA10, fosC*, *fosL1*, and *fosL2*, with all except *fosA2* and *fosA7* identified as plasmid borne [11]. Notably, all plasmid-located genes, except *fosA1* and *fosL2*, are present in *E. coli* isolates, with *fosA3* being the most commonly found variant in *E. coli* [12,13]. Plasmids that harbor *fos* genes frequently carry additional resistance genes, which facilitate the co-selection of fosfomycin resistance. Notably, *E. coli* isolates predominantly co-express *fosA3* alongside ESBL genes, such as *bla*CTX_-M_ genes, as well as various other antibiotic resistance mediated genes. Thess include genes mediating resistance to other antibiotic classes, such as aminoglycosides, β-lactams, fluoroquinolones, sulfonamides, and tetracyclines [13].

Horizontal gene transfer enables *E. coli* to acquire resistance genes, significantly contributing to its virulence and pathogenicity. Characterization of the resistance associated genes may contribute significantly to the development of advanced diagnostic approaches and more effective therapeutic strategies [14]. Further studies are necessary to determine the efficiency of fosfomycin against MDR pathogens. It is vital to identify susceptibility patterns and resistance mechanisms to guide antibiotic therapy and limit fosfomycin resistance spread.

Characterization of fosfomycin resistance mechanisms is critical because *fosA3*, in particular, undergoes efficient horizontal transmission via conjugative plasmids, necessitating systematic surveillance to prevent its dissemination among clinical isolates [15]. Therefore, this study aimed to evaluate the in vitro susceptibility to fosfomycin of clinical UPEC isolates recovered from patients with urinary tract infections at Tanta University Hospitals, Egypt, between January and December 2025, and to investigate the occurrence of plasmid-mediated *fosA3* and *fosC2* genes among fosfomycin-resistant isolates

## 2. Materials and Methods

### 2.1. Study Design and Patient Population

This prospective laboratory-based cross-sectional observational study was performed at the Department of Medical Microbiology and Immunology, Faculty of Medicine, Tanta University, between January and December 2025. The study included 158 non-duplicate clinical isolates of uropathogenic *E. coli* (UPEC) recovered from urine specimens of patients diagnosed with urinary tract infections (UTIs) who were admitted to different hospital departments and intensive care units at Tanta University Hospitals. Samples yielding other predominant microorganisms, including Gram-negative bacteria, Gram-positive bacteria, or fungi, were excluded from the analysis. All enrolled patients underwent comprehensive medical history assessment, with particular emphasis on urological symptoms, history of renal disease, associated comorbidities, prior use of antibiotics, as well as full clinical examination. The study protocol received ethical approval from the Ethics and Research Committee of the Faculty of Medicine, Tanta University (approval code: 36264PR1036/1/25), and written informed consent was obtained from all participants before their enrollment in the study.

### 2.2. Isolation and Identification of E. coli

Morning midstream urine specimens were obtained from non-catheterized patients using sterile, dry, wide-mouthed, leak-proof containers with securely fitted caps. In catheterized patients, samples were collected under strict aseptic conditions by aspirating urine from the distal portion of the catheter with a sterile syringe after prior disinfection with 70% ethyl alcohol. Urine cultures were performed on Cysteine Lactose Electrolyte Deficient (CLED) agar (HiMedia^®^, Laboratories Pvt. Ltd., Mumbai, India) using a semiquantitative calibrated loop streaking technique [16].

The diagnosis of UTI was based on the detection of ≥10^5^ colony forming units (CFU/mL) of a single microorganism in a midstream specimen, or ≥10^4^ CFU/mL in catheter-derived specimens [17]. Identification of UPEC was achieved by culturing urine samples on 5% blood agar, MacConkey and Eosin Methylene Blue agar plates (Oxoid, Basingstoke, UK). Preliminary identification relied on Gram staining and conventional biochemical testing [18]. Confirmation of isolates was achieved using the VITEK^®^ 2 automated identification system (bioMérieux, Marcy-l’Étoile, France) in accordance with the manufacturer’s guidelines. Verified UPEC isolates were preserved at −80 °C in Brain Heart Infusion broth (Biolife, Milan, Italy) supplemented with 20% glycerol (Sigma-Aldrich, St. Louis, MO, USA) for subsequent analysis.

### 2.3. Antibiotic Susceptibility Pattern of UPEC Isolates

Antimicrobial susceptibility testing was carried out using the disk diffusion method in accordance with the recommendations of the Clinical and Laboratory Standards Institute (CLSI). The antibiotics evaluated included imipenem (10 μg), meropenem (10 μg), levofloxacin (10 μg), ciprofloxacin (10 μg), norfloxacin (10 μg), gentamicin (10 μg), amikacin (30 μg), doxycycline (30 μg), tetracycline (30 μg), amoxicillin/clavulanic acid (20/10 μg), ceftriaxone (10 μg), cefotaxime (30 μg), cefepime (30 μg) aztreonam (30 μg), piperacillin/tazobactam (100/10 μg), trimethoprim–sulfamethoxazole (1.25/23.75 μg), ceftazidime avibactam (10/4 μg), and nitrofurantoin (300 μg) (Oxoid, Basingstoke, UK). Colistin susceptibility was determined using the disk elution method [19]. *E. coli* ATCC 25922 was employed as a quality control strain throughout all procedures.

Isolates exhibiting resistance to three or more antimicrobial classes were defined as multidrug-resistant (MDR). Meanwhile, isolates resistant to at least one agent in all but two or fewer antimicrobial categories—thereby remaining susceptible to only one or two classes—were classified as extensively drug-resistant (XDR) [20].

### 2.4. Phenotypic Detection of Extended-Spectrum β-Lactamase (EsβL) and Carbapenemase Producing UPEC

EsβL producing UPEC isolates were identified by the combination disc test (CDT) in accordance with CLSI guidelines. Two antibiotic discs were employed: one containing cefotaxime alone and the other containing cefotaxime combined with clavulanic acid. These were applied to Mueller–Hinton agar plates inoculated with a standardized bacterial suspension equivalent to 0.5 McFarland and incubated for 16–18 h. ESβL production was confirmed by an increase of ≥5 mm in the inhibition zone diameter around the cefotaxime–clavulanic acid disc compared to cefotaxime alone [19].

Isolates demonstrating resistance to one or more carbapenems were subsequently assessed for carbapenemase production using the modified carbapenem inactivation method (mCIM) and the EDTA-modified carbapenem inactivation method (eCIM), following CLSI recommendations [19].

### 2.5. Phenotypic Detection of Fosfomycin-Resistant UPEC Strains

The susceptibility of all UPEC isolates to fosfomycin was assessed using two approaches, in line with the recommendations of CLSI: the disk diffusion method and the agar dilution method. For disk diffusion, 200 µg fosfomycin disks supplemented with 50 µg of glucose-6-phosphate were applied on Mueller–Hinton agar (Oxoid, UK). In parallel, the minimum inhibitory concentration (MIC) was determined using the agar dilution method, where fosfomycin disodium salt (Sigma-Aldrich) was incorporated into Mueller–Hinton agar supplemented with 25 mg/L glucose-6-phosphate. Serial twofold dilutions of fosfomycin were prepared over a concentration range of 16 to 2048 mg/L [19].

### 2.6. Genotypic Detection of Plasmid Mediated Fosfomycin Resistance Genes in UPEC

Conventional PCR was utilized to identify plasmid-mediated fosfomycin resistance genes (*fosA3* and *fosC2*) among fosfomycin-resistant UPEC isolates. Plasmid DNA was extracted using the QIAGEN Plasmid Mini Kit (QIAGEN, Hilden, Germany) in accordance with the manufacturer’s guidelines. PCR amplification was carried out using the QIAGEN Taq PCR Master Mix Kit (QIAGEN, Hilden, Germany) in a total reaction volume of 25 µL. Each reaction mixture consisted of 12.5 µL of 2× Taq PCR Master Mix, 1 µL (10 pmol/µL) of each forward and reverse primer (Applied Biosystems, Foster City, CA, USA), 2 µL (100 ng/µL) of template DNA, and nuclease-free water to complete the volume. The primer sequences for each target gene are presented in Table 1.

Amplification was performed in a Rotor-Gene Q thermal cycler under the following conditions: initial denaturation at 94 °C for 3 min, followed by 35 cycles of denaturation at 94 °C for 30 s, primer annealing for 30 s (annealing temperatures for each primer are provided in Table 1), and extension at 72 °C for 1 min. A final extension step was conducted at 72 °C for 10 min, after which the reactions were held at 4 °C.

The PCR products were analyzed by electrophoresis on a 2% agarose gel stained with ethidium bromide (0.5 µg/mL). DNA fragments were visualized under ultraviolet transilluminator and documented. The presence of target genes was confirmed based on the expected amplicon sizes (Table 1) in comparison with a molecular weight DNA ladder.

## 3. Results

### 3.1. Demographic and Clinical Characteristics of Study Participants

The study included 158 patients with urinary tract infections, with a mean age of 45.7 ± 15.1 years. Diabetes mellitus was the most common comorbidity (27.8%), and 81.0% of patients reported antibiotic use within the previous three months. Detailed demographic and clinical data are presented in Table 2.

### 3.2. Antimicrobial Susceptibility Profile of UPEC Isolates

The antimicrobial susceptibility profile of the UPEC isolates demonstrated high multidrug resistance. Complete resistance was observed with amoxicillin–clavulanate and third-generation cephalosporins, whereas colistin and fosfomycin showed the highest susceptibility rates (97.5% and 90.5%, respectively). Nitrofurantoin retained moderate activity against the tested isolates. Detailed antimicrobial susceptibility data are shown in Table 3.

#### Antibiotic Resistance Profiles

**Figure 1:**
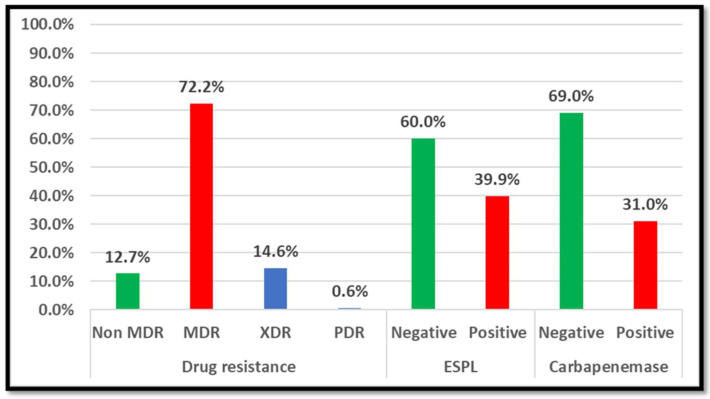
Antimicrobial resistance patterns and β-lactamase production among UPEC isolates.

In this study, the antimicrobial resistance patterns were analyzed. Most isolates were classified as multidrug-resistant (MDR), whereas extensively drug-resistant (XDR) and pandrug-resistant (PDR) phenotypes were less frequent. Furthermore, ESBL and carbapenemase production were detected among a substantial proportion of isolates. The detailed resistance phenotypes are shown in Figure 1.

### 3.3. Fosfomycin Activity Against MDR and β-Lactamase Producing Isolates

**Figure 2:**
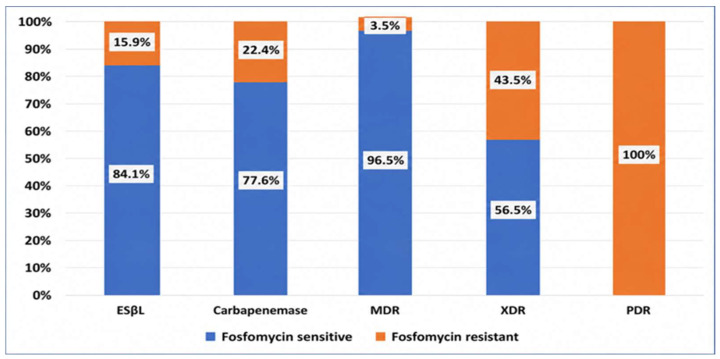
Fosfomycin activity against MDR and β-lactamase producing isolates.

Fosfomycin maintained good in vitro activity against resistant UPEC isolates, particularly among MDR isolates. The susceptibility patterns of fosfomycin against different resistance phenotypes are illustrated in Figure 2.

### 3.4. Genotypic Detection of fosA3 and fosC2 Genes in UPEC

**Figure 3:**
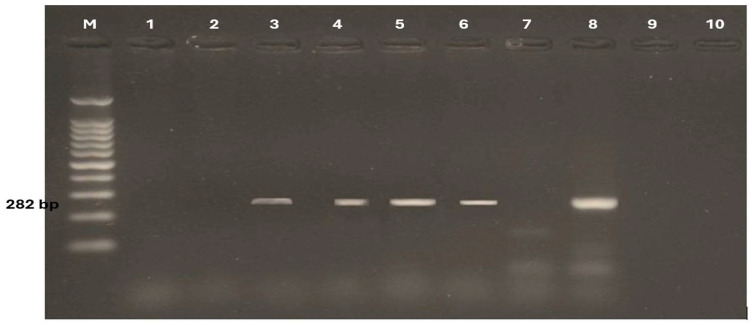
Agarose gel electrophoresis of PCR products of the *fosA3* gene amplicon among fosfomycin resistant UPEC isolates; positive isolates gave a single band of 282 bp. Lane 1: 100 pb DNA ladder marker Lane 3, 4, 5, 6, 8 positive isolates Lane 1, 2, 7, 9, 10 = *fosA3* negative isolate.

Genotypic analysis of UPEC isolates revealed that the *fosA3* gene was detected in 33.3% (5/15) of fosfomycin-resistant strains, whereas the *fosC2* gene was not identified in any of the isolates as demonstrated in Figure 3.

### 3.5. Comparison of Demographic Data and Patients’ Characteristics with Fosfomycin Resistant and Sensitive UPEC Isolates

The demographic and clinical characteristics of 158 patients infected with UPEC, categorized by the fosfomycin susceptibility of their isolates (15 resistant vs. 143 sensitive). The analysis indicates that older age and a history of urological instrumentation are significant predictors of fosfomycin resistance in this study. A highly statistically significant difference in age was observed between the two groups (*p* = 0.001). Patients with resistant isolates were significantly older, with a mean age of 55.1 ± 9.52 years, compared to 44.7 ± 15.27 years in the sensitive group. In contrast, factors such as sex, general comorbidities, and prior antibiotic use did not exhibit statistically significant differences. The study utilized a binary logistic regression model to evaluate factors predicting fosfomycin resistance, focusing on age and the presence of urinary complicating factors. Age approached significance (*p* = 0.051), while the presence of urinary complicating factors demonstrated statistical significance (*p* = 0.008), with an odds ratio of 1.732 (95% confidence interval 1.151–2.606), indicating a strong association as demonstrated in Table 4.

## 4. Discussion

Urinary tract infections (UTIs) are prevalent bacterial infections and significantly impact hospital admission and healthcare costs. The escalating issue of antimicrobial resistance has restricted the range of available treatment options, promoting a renewed interest in evaluating older antibiotics as potential solutions for this challenge [22]. In the current study, demographic analysis revealed a predominance of male patients (53.8%), which is in accordance with the findings reported by Porwal et al. and Bajracharya et al., who also reported higher proportions of hospitalized male UTI patients [23,24]. In contrast, Ehsan et al. documented a female predominance, with females comprising 79% compared to males 21% [25]. Moreover, several reports heave revealed a higher incidence of UTIs in females compared to males [26,27,28]. The mean age of enrolled cases was 45.7 ± 15.11 years (range: 18.0–85 years), which is consistent with Aref et al. who reported a comparable mean patient’s age of 45.85 ± 18.69 years in their cohort [29]. Understanding the association between underlying disease and UTIs is crucial for adequate prevention and treatment.

People with risk factors are encouraged to consult healthcare providers for appropriate preventive strategies [28]. In the present study, diabetes mellitus was the most predominant comorbidity, found in 27.8% of the participants. This was followed by cancer and chronic renal disease, each present in 20.9% of the individuals. Nearly one-quarter (22.8%) had no documented comorbidities. The majority of participants had urinary complicating factors, including the presence of Foley catheters (20.3%), nephrolithiasis (15.2%), and recent urological procedures. Additionally, 81.0% of patients reported a history of antibiotic use.

Hsiao et al. revealed that hypertension and diabetes mellitus were the most dominant comorbidities among UTI patients [30]. These findings are consistent with our findings. Moreover, Gharieb et al. found that higher rates of UTI were found in patients with urinary catheters [21].

Antimicrobial resistance is escalating, predominantly in UTIs, necessitating major research efforts [31]. Our analysis reveals that UPEC exhibits 100% resistance to cephalosporins. This is consistent with data from other sites suggesting resistance to third generation cephalosporins [25,32]. The high resistance to third generation cephalosporins suggests that empiric therapy using these agents is likely to be ineffective in our hospital. Resistance to fluoroquinolones was significant, ranging from 62.0% for levofloxacin to 75.9% for ciprofloxacin. Aligning with our results, a study by Ormeño et al. found resistance rates of 68.5% for levofloxacin and 69.6% for ciprofloxacin [33]. In Egypt, the widespread use of antibiotics for treating UTIs has contributed to significant resistance rates [34].

While the isolates retained high susceptibility to older and last-resort antibiotics, colistin appeared as the most effective agent (2.5% resistance), followed by fosfomycin (9.5% resistant), making them viable therapeutic options, underscoring the need for careful prescribing. In accordance with our results, Alshaikh et al. found that colistin and fosfomycin were the most effective antibiotics with resistance rates of 0 and 2% respectively [35]. Correspondingly, *E. coli* recovered from Mansoura Hospitals in Egypt demonstrated low resistance rates to fosfomycin (4.67%) and colistin (7.33%) as reported by El-baz et al. [36]. Furthermore, Başkan et al. observed complete susceptibility to fosfomycin among all tested isolates [37].

In contrast, Ehsan et al. reported substantially higher resistance (64%) to colistin sulfate, highlighting marked interstudy variation in this critical last-resort antibiotic [25]. The different resistance patterns of *E. coli* isolates across various geographic regions emphasize the necessity for timely local data on UPEC resistance profiles, which is essential for improving antibiotic prescribing practices, guiding empirical therapy, and formulating targeted stewardship and infection control strategies [38,39].

In this study, antimicrobial resistance patterns were assessed, revealing that 72.2% of the isolates were multidrug resistant (MDR), while 14.6% were classified as extensively drug resistant (XDR). Pandrug-resistant (PDR) isolates were infrequent, comprising only 0.6% of the total isolates. Poursina et al. discovered MDR in 73% of UPEC isolates [40], which is consistent with our findings. These data are consistent with Ahmadi et al.’s observation that 75.24% of strains were MDR, 7.62% were XDR, and 0.95% were PDR [41].

Global MDR rates in UPEC isolates vary geographically. Notably, relatively moderate prevalence rates have been reported in Nepal 64.9% [42] and China 42.38% [43], while Hungary has lower rates at 22.8% [44]. In contrast, Egypt and Iraq show significantly higher MDR rates at 90% and 99.3%, respectively [14,35]. A recent study by Başkan et al. discovered that all UPEC isolates tested were resistant to various antibiotic classes, categorizing them as MDR organisms [37].

Extended-spectrum β-lactamase (ESBL)-producing UPEC strains significantly complicate the management of UTIs. In our investigation, ESBLs were detected in 39.9% of the UPEC isolates. These results are broadly comparable to previous reports. Razaq et al. identified 45.31% of *E. coli* strains as ESBL producers [45], whereas Ahmadi et al. documented ESBL positivity in 36.19% of UPEC isolates [41]. In addition, Ehsan et al. reported that 48% of isolates were ESBL producers, further underscoring the considerable burden of ESBL mediated resistance among UPEC isolates [25].

Several studies reveal significantly high rates of ESBL-positive UPEC, contrasting with lower prevalence rates in our cohort. Naeem et al. [46] reported 57.89% from tertiary care centers, Nasir et al. [47] found 66.2% positivity, Samin et al. [48] detected 81.4%, while Khan and Bari identified 92% in UPEC isolates. These findings highlight considerable geographic and temporal variability in the prevalence of ESBL among UPEC isolates [49].

In the present study, carbapenemase production was detected in 31% of UPEC isolates, which was lower than the prevalence rate of 49% (54/110) reported by Naeem et al. in their analysis of *E. coli* isolated from urine samples [46].

Plasmid-mediated fosfomycin resistance genes, particularly *fosA* and *fosC2*, have been widely reported in Enterobacterales, with *fosA3* being the most commonly identified, especially in *E. coli* [50].

The rise in fosfomycin resistance among ESBL-producing *E. coli* has been linked to the emergence of plasmid-mediated *fosA3*. This gene was first identified in clinical *E. coli* isolates in Japan, showing a positivity rate of 1.04% [51]. Domestic animals in Asian countries are identified as sources of Enterobacterales isolates with *fosA3*, which can be transferred via conjugation. Fosfomycin resistance is more prevalent in Asia compared to Europe due to the transmission of plasmids or clones carrying *fosA3* between animals and humans [52].

In the present study, fosfomycin resistance was detected in 9.5% (15/158) of UPEC isolates, with the *fosA3* gene identified in 33.3% (5/15) of these resistant strains. No isolates harbored *fosC2* gene. These findings align with those reported by Galindo-Méndez et al. in Mexico, who observed fosfomycin resistance in 10.9% of UPEC strains. Among their resistant isolates, 36.8% (14/38) harbored *fosA3* gene [53].

Comparative studies on fosfomycin resistance rates among *E. coli* isolates have revealed significant variability across geographic regions. Bi et al. from China reported a prevalence of resistance at 6.7% (24/356), identifying the *fosA3* gene in 20 of those isolates [54]. Similarly, Bir R et al. from India found a resistance rate of 6.1% among their isolates [55]. Ibrahim and Mohammed Mosul, Iraq observed a resistance rate of 7.1% (10/140) in their isolates [14]. In contrast, Liu et al. from Taiwan documented a 4.5% resistance rate among ESBL-positive *E. coli* isolates [56]. Additionally, Khalid and Ghareeb from Baghdad, Iraq demonstrated complete susceptibility to fosfomycin in all tested isolates, highlighting the significant geographic and population-specific differences in resistance patterns [57].

In our setting, *fosA3* emerged as the predominant plasmid-mediated mechanism of fosfomycin resistance. This finding agrees with Bahy et al. (Egypt), who reported that fosfomycin resistance was primarily mediated by the plasmid-encoded gene *fosA3*, with no evidence of resistance linked to *fosC2* or *fosA* [58]. Similarly, *fosA3* was identified as the main resistance gene in previous studies [9,59,60].

Fosfomycin, an older antibacterial agent, has recently reclaimed therapeutic significance owing to the increasing prevalence of MDR Gram-negative bacterial infections, especially in UTIs. Its distinct bactericidal mode of action and absence of cross-resistance with other antibiotics classes and the ability to achieve high urinary concentrations enhance its therapeutic value against prevalent uropathogens like *E. coli* [61].

Fosfomycin is generally considered a safe antimicrobial agent, with gastrointestinal disturbances representing the most commonly reported adverse effects. The frequency of adverse events associated with oral fosfomycin ranges from 2% to 6%, with a higher incidence observed following repeated dosing [62].

The historical underutilization of fosfomycin has been attributed to the availability of newer antimicrobial agents, concerns about resistance development, limited clinical experience outside urinary tract infections, and difficulties in susceptibility testing. Nevertheless, the recent escalation in antimicrobial resistance worldwide has renewed the interest in the clinical utility and therapeutic potential of fosfomycin [63].

The determination of the mutant prevention concentration (MPC) is an in vitro approach used to evaluate the potential for antimicrobial resistance development. MPC is defined as the lowest antimicrobial concentration capable of preventing the growth of first-step resistant mutants within a large bacterial population. Maintaining drug concentrations above the MPC is considered an effective strategy for minimizing the selection and amplification of resistant bacterial subpopulations [64].

The MPC of fosfomycin against most susceptible uropathogens is considerably lower than the urinary concentrations achieved following a standard 3 g oral dose of fosfomycin tromethamine. After administration, fosfomycin is eliminated unchanged via glomerular filtration, reaching peak urinary concentrations of approximately 4000 µg/mL and maintaining levels above 100 µg/mL for up to 48 h. Consequently, urinary drug exposure remains above the MPC for an extended period, which may help suppress the emergence of resistant subpopulations and supports its clinical utility in the treatment of urinary tract infections [63,65].

In the current study, fosfomycin demonstrated susceptibility rates of 77.6%,84.1%, and 96.5% activity against carbapenemase-producing, ESBL-producing, and MDR isolates, respectively. Kaur et al. [66] similarly reported high in vitro activity of fosfomycin against UPEC with 86.9% susceptibility among MDR isolates and 88.8% among metallo-β-lactamase producers. Moreover, fosfomycin exhibited excellent activity against ESBL-producing *E. coli*., as 106/108 (98.1%) isolates were susceptible.

Similarly, Başkan et al. [37] and Gopichand et al. [67] reported complete (100%) susceptibility of fosfomycin among ESBL-producing isolates; these findings highlight the considerable in vitro activity of fosfomycin, mostly against MDR pathogens, further supporting its potential therapeutic role in the management of infections caused by resistant Gram-negative organisms. 

In the present study, analysis of 158 patients with UPEC infections revealed that older age and urological instrumentation were significant predictors of antimicrobial resistance. Patients with resistant infections had a mean age of 55.1 years, which was significantly higher than the 44.7 years observed in the susceptible group (p=0.001). In contrast, no statistically significant difference was found in sex, general comorbidities, or prior antibiotic use. By comparison, Li et al. [68] reported no significant differences observed between fosfomycin-susceptible and fosfomycin-resistant isolates in age, sex, hypertension, pulmonary infections, invasive device use, carbapenem exposure, and ICU admission. However, patients with fosfomycin-resistant infections showed a higher prevalence of immunocompromising conditions (e.g., diabetes and immunosuppressant therapy) and fungal co-infections compared to those with susceptible strains (p<0.05).

## 5. Conclusions

In our setting, fosfomycin had good in vitro activity against UPEC isolates. Therefore, fosfomycin is a promising antibiotic for the treatment of urinary tract infections caused by UPEC strains. *fosA3* emerged as the predominant plasmid-mediated mechanism of fosfomycin resistance. The detection of this transferable resistance determinant raises significant clinical and epidemiological concerns, as it may compromise the future utility of fosfomycin. Continuous surveillance, rational antibiotic stewardship, and further molecular investigations are strongly recommended to monitor the spread of *fos*-mediated resistance and to preserve the efficacy of this valuable antimicrobial agent.

## Figures and Tables

**Table 1:** PCR Primers and conditions of the studied genes.

Gene	Sequence (5′→3′)	Annealing Temperature	Amplicon Size (bp)	Reference
*fosA3*	F: GCGTCAAGCCTGGCATTT R: GCCGTCAGGGCTGAGAAA	57.5 °C	282	[21]
*fosC2*	F: TGGAGGCTACTTGGATTTG R: AGGCTACCGCTATGGATTT	50.5 °C	217	

**Table 2:** Demographic and clinical characteristics of study participants (*n* = 158).

Age		
Mean ± SD.	45.7 ± 15.11	
Min.–Max.	18.0–85.0	
	No.	%
Sex		
Male	85	53.8
Female	73	46.2
Comorbidities		
No	36	22.8
Diabetes mellitus	44	27.8
Malignancy	33	20.9
Chronic kidney disease	33	20.9
Hemodialysis	12	7.6
Urinary complicating factor		
No	64	40.5
Folly catheter	32	20.3
Recent urological intervention	24	15.2
Nephrolithiasis	24	15.2
Ureteral stent	14	8.9
Prior antibiotic use		
No	30	19.0
Yes	128	81.0

**Table 3:** Antimicrobial susceptibility profile of UPEC isolates (*n* = 158).

Antibiotic	Susceptible *n* (%)	Intermediate *n* (%)	Resistant *n* (%)
Imipenem	90 (57%)	0 (0.0%)	68 (43%)
Meropenem	90 (57%)	0 (0.0%)	68 (43%)
Gentamycin	60 (38%)	38 (24%)	60 (38%)
Amikacin	94 (59.5%)	27 (17.1%)	37 (23.4%)
Ceftazidime	0 (0.0%)	0 (0.0%)	158 (100%)
Cefepime	2 (1.3%)	0 (0.0%)	156 (98.7%)
Cefotaxime	0 (0.0%)	0 (0.0%)	158 (100%)
Aztreonam	5 (3.2%)	0 (0.0%)	153 (96.8%)
Amoxicillin–Clavulanate	0 (0.0%)	0 (0.0%)	158 (100%)
Piperacillin–Tazobactam	21 (13.3%)	3 (1.9%)	134 (84.8%)
Ceftazidime–Avibactam	70 (44.3%)	0 (0.0%)	88 (55.7%)
Trimethoprim–Sulfamethoxazole	53 (33.5%)	3 (1.9%)	102 (64.6%)
Tetracycline	64 (40.5%)	7 (4.4%)	87 (55.1%)
Doxycycline	91 (57.6%)	26 (16.5%)	41 (25.9%)
Norfloxacin	51 (32.3%)	6 (3.8%)	101 (63.9%)
Ciprofloxacin	29 (18.4%)	9 (5.7%)	120 (75.9%)
Levofloxacin	44 (27.8%)	16 (10.1%)	98 (62%)
Nitrofurantoin	106 (67.1%)	3 (1.9%)	49 (31%)
Fosfomycin	143 (90.5%)	0 (0.0%)	15 (9.5%)
Colistin	154 (97.5%)	0 (0.0%)	4 (2.5%)

**Table 4:** Comparison of demographic data and patients’ characteristics with fosfomycin resistant and sensitive UPEC isolates.

	Resistant (*n* = 15)		Sensitive (*n* = 143)		Test of Sig.	*p*
Age					t 3.763	0.001 *
Mean ± SD.	55.1 ± 9.52		44.7 ± 15.27			
Min.–Max.	35.0–65.0		18.0–85.0			
	No.	%	No.	%		
Sex					χ^2^ 2.545	0.111
Male	11	73.3	74	51.7		
Female	4	26.7	69	48.3		
Comorbidities					χ^2^ 7.291	pMC 0.120
No	2	13.3	34	23.8		
Diabetes mellitus	2	13.3	42	29.4		
Malignancy	6	40.0	27	18.9		
Chronic kidney disease	5	33.3	28	19.6		
Hemodialysis	0	0.0	12	8.4		
Urinary complicating factor					χ^2^ 23.242	pMC <0.001 *
No	2	13.3	62	43.4		
Folly catheter	1	6.7	31	21.7		
Recent urological intervention	6	40.0	18	12.6		
Nephrolithiasis	1	6.7	23	16.1		
Ureteral stent	5	33.3	9	6.3		
Prior Antibiotic use					χ^2^ 3.884	pFE 0.076
No	0	0.0	30	21.0		
Yes	15	100.0	113	79.0		

t: Independent *t* test, χ^2^: Chi square test, MC: Monte Carlo Exact, * *p* ≤ 0.05 (statistically significant).

## Data Availability

The original contributions presented in this study are included in the article. Further inquiries can be directed to the corresponding authors.
